# A New Base for Artificial Teeth

**Published:** 1852-04

**Authors:** 


*A New Base for Artificial Teeth.*—Mr. John Spence, surgeon dentist of Edinburgh, Scotland, gives in a letter to the senior editor, the following description of a set of under teeth which he made in the summer of 1851, for a gentleman, "who, owing to the great absorption of the alveoli, required an unusual depth of socket, and whose gums were very easily irritated. He has been," says Mr. S., "my patient for many years, and I have tried him with mineral teeth set on gold, and also in hippopotamus tusk—the former was too irritating to the gums, the latter soon became decomposed and offensive from the vitiated state of his saliva, and required to be renewed every twelve or eighteen months. Under these circumstances, I proposed making the base of marble, with ten mineral teeth set into it; this, he gladly agreed to try, and having used them since July, he declares they are the most pleasant and most efficient he has ever yet had.

"I have since made another set, which is also giving equal satisfaction, but it has been about half the time in use. I may mention that both patients have upper sets on gold, with spiral springs attached to the under marble pieces.

"After trying several kinds, the marble I found best is the *Gristola de Carara;* it is hard without being brittle, and takes a high polish. The teeth I stained a little yellowish, and the gum a pale pink, but I fear the acid of the saliva, particularly of the first patient, will soon eat it out, and I question, if in time, it will not even eat into the marble itself."

The advantages derived in these cases from the use of marble, might be obtained by cast block tin bases, prepared in the manner as described by Dr. E. G. Hawes, of New York, in a former number of the Journal, which is not like marble, liable to be broke by a fall. There are many cases where weight and thickness of base are very desirable, if not absolutely necessary for artificial teeth for the lower law.

